# Idiopathic Normal Pressure Hydrocephalus and Elderly Acquired Hydrocephalus: Evaluation With Cerebrospinal Fluid Flow and Ventricular Volume Parameters

**DOI:** 10.3389/fnagi.2020.584842

**Published:** 2020-10-30

**Authors:** Wen-Jie He, Xi Zhou, Jia Long, Qi-Zhong Xu, Xian-jian Huang, Jun Jiang, Jun Xia, Guang Yang

**Affiliations:** ^1^Department of Radiology, The First Affiliated Hospital of Shenzhen University, Health Science Center, Shenzhen University, Shenzhen, China; ^2^Department of Radiology, Shenzhen Second People’s Hospital, Shenzhen, China; ^3^Department of Neurosurgery, The First Affiliated Hospital of Shenzhen University, Health Science Center, Shenzhen University, Shenzhen, China; ^4^Department of Neurosurgery, Shenzhen Second People’s Hospital, Shenzhen, China; ^5^National Heart and Lung Institute, Faculty of Medicine, Imperial College London, London, United Kingdom

**Keywords:** elderly acquired hydrocephalus, idiopathic normal pressure hydrocephalus, aqueductal stroke volume, cerebrospinal fluid flow, phase-contrast MRI, ventricular volume

## Abstract

**Purpose:**

To investigate differences in cerebrospinal fluid (CSF) flow through the aqueduct and to determine whether there is a relationship between CSF flow and ventricular volume parameters in idiopathic normal pressure hydrocephalus (iNPH) patients, elderly acquired hydrocephalus patients and age-matched healthy volunteers by phase-contrast MR (PC-MR).

**Methods:**

A total of 40 iNPH patients and 41 elderly acquired hydrocephalus patients and 26 age-matched healthy volunteers in the normal control (NC) group were included between November 2017 and October 2019 in this retrospective study. The following CSF flow parameters were measured with PC-MR: peak velocity, average velocity (AV), aqueductal stroke volume (ASV), net ASV, and net flow. The following ventricular volume parameters were measured: ventricular volume (VV), brain volume, total intracranial volume, and relative VV. Differences between the iNPH and acquired hydrocephalus groups were compared Mann–Whitney *U* test and correlations between CSF flow and ventricular volume parameters were assessed using the Spearman correlation coefficient.

**Results:**

Aqueductal stroke volume was significantly higher in the iNPH and acquired hydrocephalus groups than in the NC group, but did not differ significantly between the iNPH group and acquired hydrocephalus group. The AV, net ASV, and net flow in the iNPH and acquired hydrocephalus groups were significantly higher than those in the NC group (*P* < 0.0001), and those in the acquired hydrocephalus group were significantly higher than those in the iNPH group (*P* = 0.01, *P* = 0.007, *P* = 0.002, respectively). The direction of the AV and net ASV significantly differed among the three groups. There were no associations between the volume parameters and CSF flow according to PC-MR among the three groups.

**Conclusion:**

Compared with iNPH, elderly acquired hydrocephalus demonstrated higher CSF hyperdynamic flow. Although increased CSF flow may contribute to further changes in ventricular morphology, there is no linear relationship between them. These findings might help increase our understanding of flow dynamics in iNPH and elderly acquired hydrocephalus.

## Introduction

Normal pressure hydrocephalus (NPH), first reported by Adams et al. (1965), is a treatable syndrome with a triad of symptoms comprising gait instability, cognitive disturbances, urinary incontinence and the presence of normal cerebrospinal fluid (CSF) pressure on lumbar puncture. The characteristic radiologic finding is communicating hydrocephalus, and symptom improvements can be found after implantation of a CSF shunt (Relkin et al., 2005; Mori et al., 2012). NPH is classified into idiopathic NPH (iNPH) of unknown etiology and secondary NPH (sNPH) of known etiology; causes of sNPH include subarachnoid hemorrhage, meningitis, and trauma (Relkin et al., 2005; Mori et al., 2012). Recently, the term of sNPH has been mostly abandoned and classified as “acquired hydrocephalus” (Williams et al., 2019). Specifically, sNPH was classified as a kind of elderly acquired communicating hydrocephalus according to etiology and classification (Williams et al., 2019; Karimy et al., 2020).

Phase-contrast MR (PC-MR) is a non-invasive method that is widely used for measuring CSF flow (dynamics) at the level of the aqueduct, by synchronizing the acquisition of images with the cardiac cycle (Bradley et al., 1996; Ringstad et al., 2015, 2016). Previous studies used PC-MRI to demonstrate that patients with iNPH presented net retrograde aqueductal flow and hyperdynamic flow of CSF (Ringstad et al., 2016; Yin et al., 2017). Several CSF parameters measured with PC-MR, including both aqueductal stroke volume (ASV) and net ASV, may play an important role in the pathophysiology of ventriculomegaly in iNPH patients (Bradley et al., 1996; Ringstad et al., 2016; Yin et al., 2017). However, much of the literature focuses on iNPH and less on acquired hydrocephalus. CSF flow has been underexplored in patients with acquired hydrocephalus than in patients with iNPH. It is unclear whether CSF flow in iNPH and acquired hydrocephalus patients is comparable. In addition, ventricular enlargement is a key diagnostic criterion for hydrocephalus, but it remains unclear whether the increase in CSF flow stems from or causes the changes in ventricular morphology that are observed in iNPH and acquired hydrocephalus (Chiang et al., 2009; Yin et al., 2017). Meanwhile, CSF flow dynamics parameters measured in the cerebral aqueduct may be partly age and sex dependent (Sartoretti et al., 2019).

Thus, the purpose of this study was to investigate differences in CSF flow through the aqueduct among iNPH patients, elderly acquired hydrocephalus patients and age matched healthy volunteers by using PC-MRI. Furthermore, this study also aimed to determine whether there is a relationship between CSF flow and ventricular volume in iNPH patients, acquired hydrocephalus patients and healthy volunteers.

## Materials and Methods

### Study Participants

**FIGURE 1:**
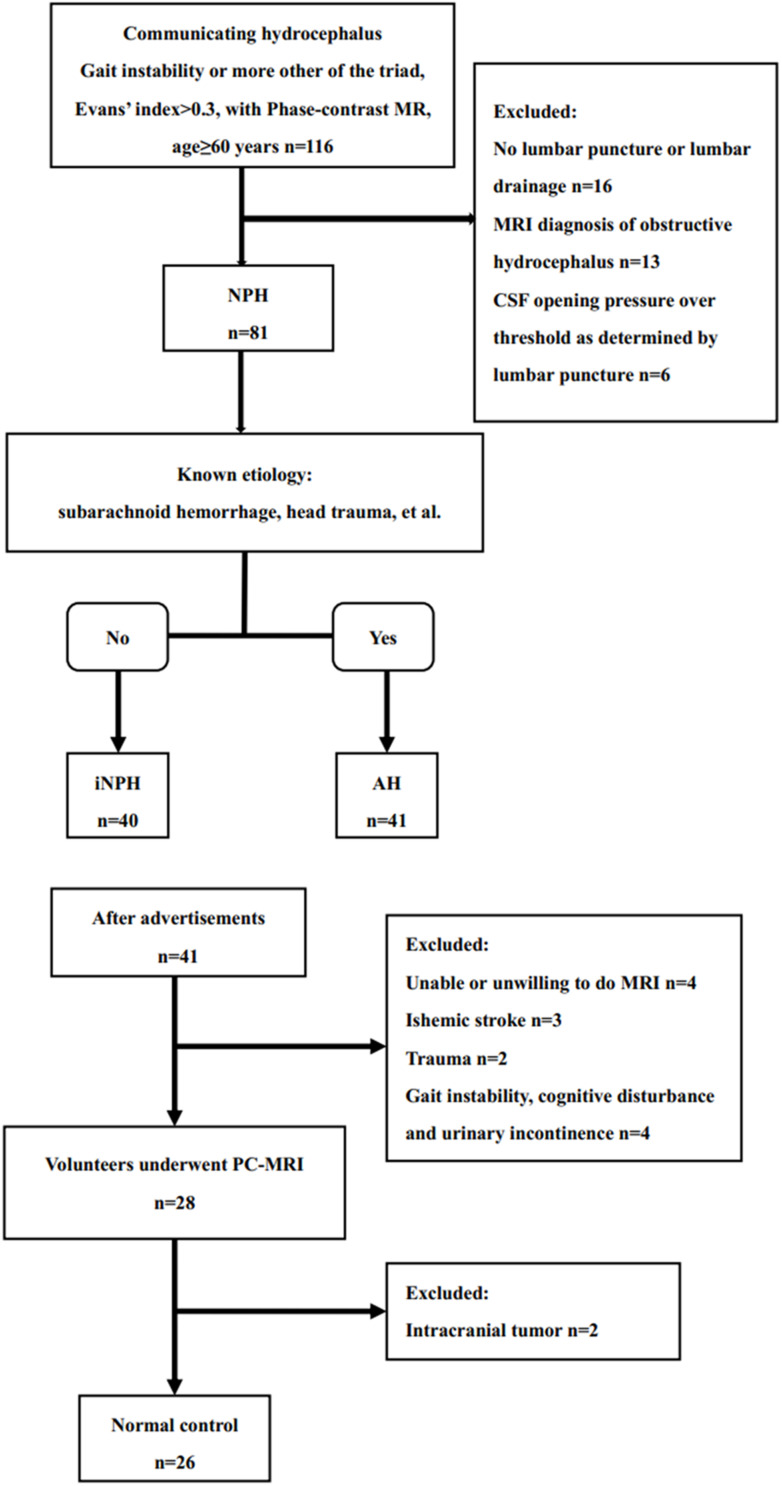
Study flow chart for the inclusion of participants in the iNPH, acquired hydrocephalus and normal control groups from the initial screening to the final analysis.

This study comprised three groups: the normal control (NC) group, iNPH group, and acquired hydrocephalus group. Figure 1 depicts the flow chart for the inclusion of patients in these three groups from the initial screening to the final analysis. Data from 116 consecutive elderly patients (60–86 years) with communicating hydrocephalous using PC-MR admitted to our hospital between November 2017 and October 2019 were retrospectively reviewed. Gait disturbance is generally considered to be the principal symptom of NPH and the parameter most likely to improve with shunt surgery (Relkin et al., 2005; Mori et al., 2012); hence, we chose gait impairment as the primary criterion for verifying shunt and CSF tap test response. On the basis of the iNPH guidelines and the outcome of the CSF tap test within the department of neurosurgery (Relkin et al., 2005; Mori et al., 2012), 40 patients fulfilled the diagnosis of possible iNPH, 41 patients were diagnosed with acquired hydrocephalus that developed after known etiology, such as history of prior meningitis, trauma, intracranial hemorrhage, prior neurosurgery. 41 acquire hydrocephalus suffered from different types of etiologies: five patients had aneurysmal subarachnoid hemorrhage; 13 patients had intracranial hemorrhage; 19 patients had trauma, of which 16 patients underwent cranioplasty; three patients had prior neurosurgery because of brain tumor; one patient had meningitis.

To recruit age matched healthy elderly volunteers for the NC group, advertisements were placed on several local community bulletin boards asking elderly persons (60–90 years) to apply for participation in the study if they considered themselves healthy. After telephone interviews conducted by two co-authors, the NC group comprised 41 age-matched healthy participants during the same time period as the iNPH patients. Four participants were excluded because of suspected cognitive disorder, unexplained gait disturbances, urinary urgency or urinary incontinence, five because of a previous history of ischemic stroke or trauma, and four because of unwilling to do MR. Atrophy, lacunar strokes, transient dizziness, and headache were not used as exclusion criteria. twenty-six age-matched and gender-matched healthy volunteers were recruited. The protocol was approved by our Hospital Bioethics Committee (approval no. KS20190114001).

### MRI Sequence

The participants in all three groups underwent PC-MRI examination with the same scanning sequence. In order to reduce heterogeneities in baseline aqueductal flow with PC-MR as much as possible, PC-MR scanning time of all patients with hydrocephalus and participants of NC group were selected at night (8–10 p.m) (Blitz et al., 2018) NE. Ref. All MRI images were obtained using a 3.0T MRI scanner (Prisma, Siemens, Erlangen, Germany) with 20-channel phase-array head coils. All patients and volunteers underwent 3D-T1WI and a retrospective cardiac-gated phase-contrast CSF flow quantification sequence. Sagittal 3D-T1WI scans were performed with a magnetization-prepared rapid acquisition gradient-echo sequence, which covered the whole head. The sequence parameters were as follows: TR/TE = 2300/2.98 ms; flip angle = 9°; slice thickness = 1 mm; field of view, 256 mm × 256 mm; matrix, 256 × 256; and pixel size, 1 mm × 1 mm. The acquisition parameters for PC-MRI were as follows: TR/TE = 21/7 ms; field of view, 160 mm × 160 mm; slice thickness/slice intervals, 6/1.2 mm; velocity encoding, 20 cm/s, which was increased to 25 cm/s if aliasing occurred; acquisition time, ∼183 s; and flip angle, 10°. In the transverse acquisition plane, the direction of encoding of the flow direction was performed from the feet to the head. During diastole, CSF flows in the velocity-encoded cine direction from the feet to the head, meaning forward flow. During systole, CSF flows in the velocity-encoded cine direction from the head to the feet, meaning backward flow. Positive values represent the caudocranial direction, and negative values represent the craniocaudal direction.

### Imaging Analysis

**FIGURE 2:**
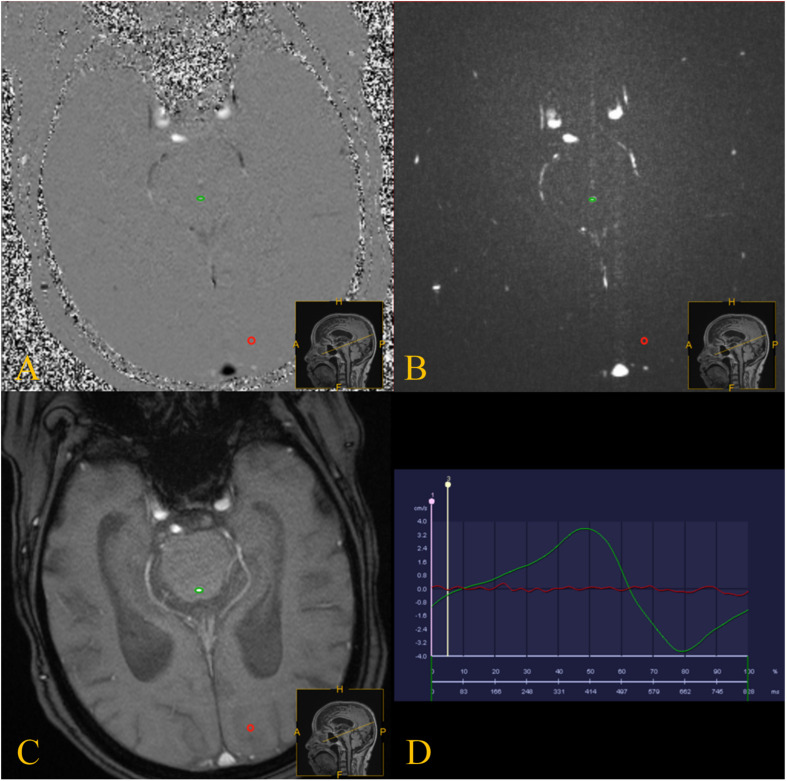
PC-MRI was performed with the slice orientation perpendicular to the aqueduct. **(A–C)** PC-MRI with a manually drawn region of interest (green circle) defining the aqueduct, and the reference region of interest is shown by the red circles. **(D)** the red line represents the flow of the reference region of interest, and the green line represents the CSF flow though the aqueduct.

Quantitative analysis of CSF flow parameters was performed using Flow Quantification software provided with the MR scanner. The region of interests was manually defined along the outer border of the aqueduct by an experienced neuroradiologist who has 15 years of experience in brain MRI interpretation (Figure 2).

Cerebrospinal fluid flow in the craniocaudal direction was defined as antegrade flow. The peak velocity, average velocity (AV), ASV, net ASV, and net flow were measured. The ASV was defined as the mean volume of CSF flowing craniocaudally during systole and caudocranially during diastole (Bradley, 2015). The net ASV was calculated by subtracting the PC-MRI-derived volumetric estimate of retrograde aqueductal flow from that of antegrade flow during one cardiac cycle (Ringstad et al., 2016; Yin et al., 2017). Net flow (mL/min) represents the net CSF aqueductal flow rate per minute and was calculated by multiplying the net ASV by the heart rate (Ringstad et al., 2016), which was measured using the finger pulse trigger of the MRI scanner.

The quantitation of ventricular volume parameters was assessed using 3D Slicer software (Surgical Planning Laboratory, Brigham and Women’s Hospital, Boston, MA, United States). The ventricular volume parameters comprised ventricular volume (VV), brain volume (the volume of brain parenchyma), sulcal volume and total intracranial volume. Relative VV was measured as the ratio of VV to total intracranial volume. For the analysis of ventricular dilation relative to the sulcal spaces, the VV was divided by the sulcal volume (Ringstad et al., 2016). Two independent operators blindly measured the ventricular volume parameters.

### Statistical Analysis

All statistical analyses were performed using SPSS 24 (IBM Corporation, Armonk, NY, United States). The Kruskal–Wallis test was performed to compare the differences in the measured values among groups. Differences between the iNPH and acquired hydrocephalus groups were determined with the Mann–Whitney *U* test. Correlations between CSF flow parameters and ventricular volume parameters were determined with the Spearman correlation coefficient. A chi-square test was performed to assess the difference in the CSF flow direction among the iNPH group, acquired hydrocephalus group and NC group. Statistical significance was accepted at the 0.05 level (two-tailed).

## Results

The demographic information, volume parameters and PC-MR CSF flow results of the iNPH patients, acquired hydrocephalus patients and NC groups are presented in Table 1. There was no significant difference among the three groups regarding age or gender. The mean time from primary etiology to hydrocephalus in acquired hydrocephalus patients was 47.5 days (47.5 ± 34.6 days, range = 15–180 days). The average duration of symptoms in iNPH patients was 3 years (3.0 ± 1.8 years, range = 0.5–7 years).

**TABLE 1:** Demographic information, volume parameters and PC-MRI CSF flow of the iNPH patients, acquired hydrocephalus and healthy volunteers.

	iNPH	AH	healthy	iNPH vs. AH vs. healthy significance	iNPH vs. AH significance
Demographic information					
Number (N)	40	41	26		
Age (years)	70.8 ± 6.6	68.6 ± 5.1	67.2 ± 6.2	NS	NS
Gender (female/male)	22/18	18/23	13/13	NS	NS
Heart rate (bpm)	81.0 ± 22.3	83.8 ± 17.9	79.1 ± 14.22	NS	NS
Volume parameters					
Ventricular volume (cmł)	124.4(21.3)	172.0(64.5)	40.1(14.6)	*P* < 0.0001	*P* = 0.007
Brain volume (cmł)	986.8(199.7)	1049.2(228.4)	1013.4(205.6)	NS	NS
Sulcal volume (cmł)	477.1(180.6)	539.5(107.9)	391.6(89.4)	*P* < 0.0001	NS
Total intracranial volume (cmł)	1479.8(287.7)	1632.4(267.0)	1408.3(163.8)	*P* < 0.0001	*P* = 0.011
Ventricular volume/Sulcal volume (%)	25.4(13.4)	32.0(9.5)	10.2(8.2)	*P* < 0.0001	NS
Relative ventricular volume (%)	8.9(2.9)	10.8(3.0)	3.5(1.8)	*P* < 0.0001	*P* = 0.019
PC-MR CSF flow					
ASV (mL)	0.0195(0.0435)	0.0270(0.0585)	0.015(0.0108)	*P* = 0.027	NS
net ASV (mL)	0.0075(0.013)	0.0120(0.045)	0.0025(0.004)	*P* < 0.0001	*P* = 0.007
Antegrade-directed net ASV (N)	5	14	24	*P* < 0.0001	*P* = 0.022
Retrograde-directed net ASV (N)	35	27	2		
Average velocity (cm/s)	0.2425(0.5665)	0.4390(0.5680)	0.138(0.1501)	*P* < 0.0001	*P* = 0.010
Antegrade-directed average velocity (N)	7	14	22	*P* < 0.0001	NS
Retrograde-directed average velocity (N)	33	27	4		
Peak Velocity (cm/s)	5.374(7.158)	6.641(8.760)	5.8105(3.760)	NS	NS
Antegrade-directed peak velocity (N)	26	18	16	NS	NS
Retrograde-directed peak velocity (N)	14	23	10		
Net flow volume (mL)	0.5625(0.977)	1.000(4.0835)	0.188(0.314)	*P* < 0.0001	*P* = 0.002

*iNPH, idiopathic normal pressure hydrocephalus; AH, acquire hydrocephalus; ASV, aqueductal stroke volume; a Significance determined by Kruskal–Wallis test and Mann–Whitney *U* test (NS, non-significant).*

The intraclass correlation coefficients were 0.97, 0.96, 0.98, and 0.99 for the VV, brain volume, sulcal volume, and total intracranial volume, respectively. In terms of volume parameters, the VV, total intracranial volume, and relative VV in the iNPH group and acquired hydrocephalus group were significantly higher than those in the NC group (*P* < 0.0001), and those in the acquired hydrocephalus group were significantly higher than those in the iNPH group (*P* = 0.007, *P* = 0.011, *P* = 0.019, respectively). The sulcal volume and VV/sulcal volume in the iNPH group and acquired hydrocephalus group were also significantly higher than those in the NC group (*P* < 0.0001), although in the case of these two parameters, the iNPH group and acquired hydrocephalus group did not significantly differ. The brain volume (the volume of brain parenchyma) did not differ significantly among the three groups.

**FIGURE 3:**
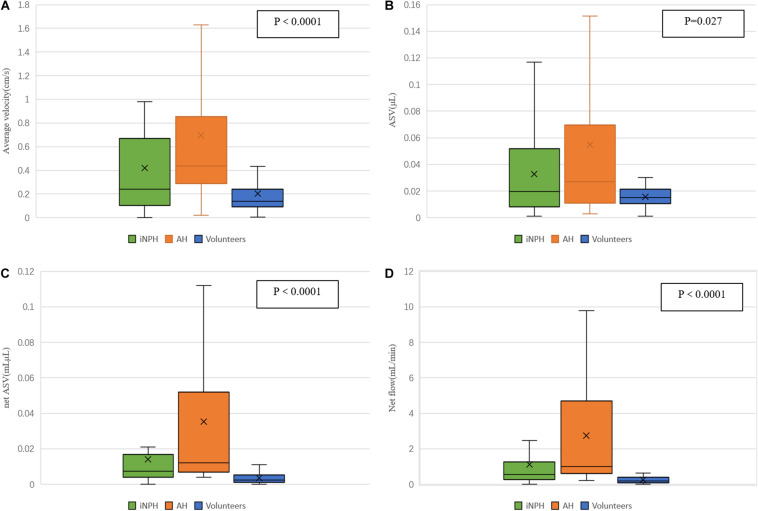
Differences in estimated CSF flow parameters in the aqueduct among iNPH patients, acquired hydrocephalus patients and healthy volunteers. **(A)** the volume of the AV in the aqueduct; **(B)** the ASV in the aqueduct; **(C)** the net ASV in the aqueduct; **(D)** the minute flow volume in the aqueduct.

The ASV was significantly higher in the iNPH and acquired hydrocephalus groups than in the NC group but did not differ significantly between the iNPH group and acquired hydrocephalus group. The AV, net ASV and net flow in the iNPH group and acquired hydrocephalus group were significantly higher than those in the NC group (*P* < 0.0001) (Figure 3), and those in the acquired hydrocephalus group were significantly higher than those in the iNPH group (*P* = 0.01, *P* = 0.007, *P* = 0.002, respectively).

**FIGURE 4:**
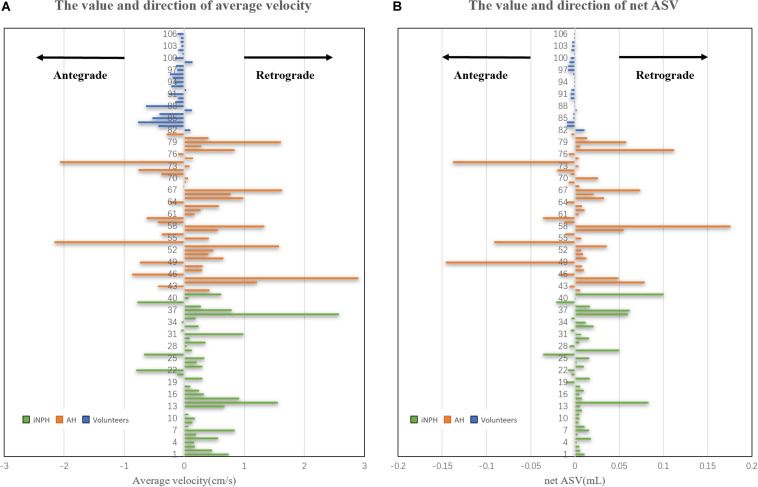
Estimated CSF net ASV and AV in the cerebral aqueduct of iNPH patients, acquired hydrocephalus patients and healthy volunteers. **(A)** the volume and direction of the AV in the cerebral aqueduct. **(B)** the volume and direction of the net ASV in the cerebral aqueduct.

The peak velocity and the direction of the peak velocity did not differ significantly among the three groups. There was a significant difference in the direction of the AV among the iNPH group, acquired hydrocephalus group and NC group (*P* < 0.0001). However, we found that the direction of the AV was not significantly different between the iNPH group and acquired hydrocephalus group. The direction of the net ASV was caudocranial in 35 iNPH patients (35/40) and 27 acquired hydrocephalus patients (27/41) and was craniocaudal in 15 healthy volunteers (15/20) (Figure 4), revealing significant differences among the three groups (*P* < 0.001). Finally, the direction of the net ASV was not significantly different between the iNPH group and the acquired hydrocephalus group.

There were no associations between the volume parameters (VV, brain volume, sulcal volume, total intracranial volume, VV/sulcal volume, and relative VV) and CSF flow parameters (peak velocity, AV, ASV, net ASV, and net flow) in the iNPH group, acquired hydrocephalus group or NC group (Table 2).

**TABLE 2:** Relationship between CSF flow and volume parameters.

CSF flow Volume parameters		ASV *r*(*P*)	net ASV *r*(*P*)	Average velocity *r*(*P*)	Peak velocity *r*(*P*)	Net flow volume *r*(*P*)
Ventricular volume	iNPH group	0.09(0.971)	0.156(0.535)	0.117(0.645)	−0.133(0.598)	0.158(0.531)
	AH group	−0.139(0.518)	0.176(0.410)	0.058(0.787)	−0.291(0.167)	0.157(0.465)
	NC group	−0.112(0.476)	0.202(0.195)	0.083(0.598)	−0.184(0.236)	0.203(0.192)
Brain volume	iNPH group	0.049(0.848)	−0.011(0.964)	0.149(0.556)	−0.112(0.657)	−0.061(0.810)
	AH group	0.276(0.191)	0.489(0.201)	0.058(0.787)	−0.291(167)	0.157(0.465)
	NC group	0.096(0.541)	0.242(0.1170	0.153(0.329)	−0.067(0.670)	0.247(0.110)
Sulcal volume	iNPH group	−0.049(0.848)	0.102(0.688)	−0.063(0.804)	−0.030(0.906)	0.116(0.648)
	AH group	0.018(0.932)	−0.119(0.580)	−0.195(0.362)	0.011(0.958)	−0.168(0.433)
	NC group	0.092(0.557)	0.061(0.699)	−0.046(0.770)	0.44(0.778)	0.220(0.891)
Total intracranial volume	iNPH group	−0.020(0.938)	0.537(0.822)	−0.208(0.409)	−0.164(0.515)	0.020(0.938)
	AH group	0.339(0.105)	0.422(0.140)	0.223(0.296)	0.092(0.668)	0.406(0.094)
	NC group	0.173(0.267)	0.298(0.053)	−0.041(0.796)	0.127(0.417)	0.279(0.070)
Ventricular volume/Sulcal volume	iNPH group	−0.120(0.527)	0.041(0.830)	−0.278(0.137)	0.019(0.922)	0.081(0.670)
	AH group	−0.271(0.148)	0.015(0.938)	−0.339(0.067)	0.070(0.714)	0.020(0.916)
	NC group	0.163(0.389)	0.025(0.896)	0.300(0.108)	0.084(0.659)	0.035(0.853)
Relative ventricular volume	iNPH group	0.036(0.887)	0.120(0.635)	−0.148(0.559)	−0.055(0.829)	0.117(0.645)
	AH group	−0.318(0.130)	−0.100(0.643)	0.172(0.421)	0.387(0.062)	−0.110(0.610)
	NC group	−0.210(0.177)	0.055(0.725)	−0.021(0.895)	−0.225(0.147)	0.065(0.678)

*AH, acquired hydrocephalus; ASV, aqueductal stroke volume; r, correlation coefficient; P, *p* value.*

## Discussion

In this study conducted in iNPH patients, acquired hydrocephalus patients and healthy volunteers, a main observation was that most CSF flow parameters except peak velocity were higher in the NPH groups than in NC group. The directions of the AV and net ASV in NPH groups were retrograde flow and opposite to that of the NC group. On the other hand, there were no associations between the volume parameters and CSF flow based on PC-MRI in those groups.

We found that AV, ASV, net ASV, and net flow volume in the NPH groups were significantly higher than those in the NC group, and those in the acquired hydrocephalus group except ASV were significantly higher than iNPH group. ASV has previously been advocated as a biomarker for the selection of patients for differential diagnosis in iNPH (Bradley et al., 1996). However, we did not find a significant difference in the ASV between iNPH and acquired hydrocephalus patients. In fact, the reliability of the ASV is controversial (Ringstad et al., 2016; Blitz et al., 2018). The results of the above parameters reflected the hyperdynamic CSF flow in iNPH and acquired hydrocephalus patients and indicated that acquired hydrocephalus patients had higher hyperdynamic flow than iNPH patients. Previous studies have demonstrated that increased CSF flow was attributed to decreased intracranial compliance (Wagshul et al., 2006; Penn et al., 2011). Intracranial compliance represents a bridge between blood flow into the cranium and CSF flow into the ventricles, and it depends on CSF flow oscillations in the cranial subarachnoid space and vascular compliance (Yin et al., 2017).

In addition, CSF production and flow are not limited to the classic CSF circulation theory, which suggested that CSF is produced by the choroid plexus and reabsorbed by the arachnoid villi. The brain parenchyma and “glymphatic system” play an important role in the production, clear and flow of CSF and interact with intracranial compliance (Iliff et al., 2012; Igarashi et al., 2014). As a result of secondary etiology, such as trauma, intracranial hemorrhage, and prior neurosurgery, the brain parenchyma, “glymphatic system” and arachnoid environment was rapidly damaged to varying degrees in acquired hydrocephalus patients, and intracranial compliance decreases rapidly, resulting in hyperdynamic CSF flow. Interestingly, in our study, we found that the decreased intracranial compliance was relatively rapid, and ventricular dilatation is relatively slow. Yin et al. (2017) reported that a decrease in intracranial compliance occurs before ventricular enlargement in iNPH. Our study showed that the time of ventricular enlargement in acquired hydrocephalus was significantly shorter than that in iNPH. Therefore, we infer that the change in intracranial compliance is faster and more dramatic in acquired hydrocephalus than in iNPH, resulting in ventricular enlargement within a few weeks or months.

Furthermore, we found the direction of both the AV and net ASV in most acquired hydrocephalus and iNPH patients was caudocranial, that was, retrograde. In contrast, the direction of these parameters in most of the NC group was craniocaudal (antegrade). Previous studies have also found similar results using PC-MR (Ringstad et al., 2015, 2016; Blitz et al., 2018). Most of the iNPH patients with reversal of aqueductal flow had signs of reduced intracranial compliance at the same time, and retrograde aqueductal flow (the positive pressure gradient from the ventricles to the parenchyma) can be hypothesized to facilitate the increase in ventricular size (Ringstad et al., 2016; Lindstrom et al., 2018). Ng et al. (2009) suggested that ventricular enlargement is a symptom rather than a cause of the triad of symptoms. We find that the reversed CSF pressure gradient in acquired hydrocephalus is similar to those in iNPH. Based on the decreased intracranial compliance and net retrograde flow mentioned above, we speculate that in both iNPH and acquired hydrocephalus, the decrease in intracranial compliance is what leads to the increase in CSF dynamics and the change in flow direction (by changing the direction of the pressure gradient), leading to ventricular enlargement in the brain.

Besides, VV, total intracranial volume, VV/sulcal volume and relative VV in acquired hydrocephalus patients, whose CSF hyperdynamic flow was higher than iNPH patients, were all significantly higher than those in iNPH patients in this study. In some acquired hydrocephalus patients, the increase in total intracranial volume was due to a change in volume after skull removal due to trauma. Compared with iNPH, acquired hydrocephalus has larger volume parameters and higher CSF hyperdynamic flow. We suggest that increased CSF flow may further increase changes in ventricular morphology.

However, we did not reproduce previous findings of correlations between CSF parameters and volume parameters, such as between ASV and VV, in any of the three groups (Chiang et al., 2009; Ringstad et al., 2015). A previous study reported an association between ASV and VV, but the result was based on a small cohort of patients, some of whom may not have had iNPH (Chiang et al., 2009). The degree of ventricular enlargement in NPH might be influenced by multiple factors, not a single factor or linear relationship (Crook et al., 2020).

Our study has some limitations. All of the participants were recruited at a single center. This study was a retrospective study and lacks prospective analysis. We did not assess the response of iNPH or acquired hydrocephalus patients to shunting, because some patients have not been followed up for 1 year and some acquired hydrocephalus patients were lost to follow-up after shunting. This is a preliminary study and the following studies should focus on whether PC-MR parameters could predict the outcome of the shunt in iNPH or acquired hydrocephalus.

## Conclusion

In conclusion, compared with iNPH patients, acquired hydrocephalus patients demonstrated higher CSF hyperdynamic flow, with increased CSF flow and volume parameters. Although increased CSF flow may contribute to further changes in ventricular morphology, there is no linear relationship between them. These findings might help increase our understanding of flow dynamics in iNPH and acquired hydrocephalus, as reduced intracranial compliance and reversal of aqueductal flow can be hypothesized to facilitate the increase in ventricular size.

## Data Availability Statement

The raw data supporting the conclusions of this article will be made available by the authors, without undue reservation.

## Ethics Statement

The studies involving human participants were reviewed and approved by the Ethics Committee of The First Affiliated Hospital of Shenzhen University and Shenzhen Second People’s Hospital. The participants provided their written informed consent to participate in the study.

## Author Contributions

W-JH performed the experiments, analyzed the data, and wrote the original draft of the manuscript. JJ and JL performed the experiments, analyzed the data, and revised the manuscript. XZ contributed to the experiments. Q-ZX contributed to the data analysis. X-H contributed to the manuscript revision. GY contributed to the experiment design and manuscript revision. JX designed the project, supervised the experiments, drafted and revised the manuscript. All authors read and approved the final manuscript.

## Conflict of Interest

The authors declare that the research was conducted in the absence of any commercial or financial relationships that could be construed as a potential conflict of interest.
